# Morphological Variations and Anomalies of the Sella Turcica on Lateral Cephalograms of Cleft-Palate-Only (CPO) Patients

**DOI:** 10.3390/diagnostics13152510

**Published:** 2023-07-27

**Authors:** Alicja Zawiślak, Agnieszka Jankowska, Katarzyna Grocholewicz, Joanna Janiszewska-Olszowska

**Affiliations:** 1 Department of Maxillofacial Orthopaedics and Orthodontics, Institute of Mother and Child, 01-211 Warsaw, Poland; alizawislak@imid.med.pl; 2 Department of Interdisciplinary Dentistry, Pomeranian Medical University in Szczecin, 70-111 Szczecin, Poland; katarzyna.grocholewicz@pum.edu.pl; 3 Private Practice “Dental Clinic Jankowscy”, 68-200 Żary, Poland; agnieszkajankowska2301@gmail.com

**Keywords:** cleft palate, sella turcica, cephalometry

## Abstract

Sella turcica abnormalities were reported in malocclusions and clefts. No studies were found on sella turcica abnormalities in CPO patients. This study aimed to compare the prevalence of sella turcica abnormalities on cephalometric radiographs in CPO versus non-cleft orthodontic patients. Cephalograms of CPO patients (*n* = 89) and controls (*n* = 89) were analyzed for normal sella turcicae and sella turcica abnormalities. Then, cephalometric analysis was performed using specialized software. Statistical analysis was performed using the Rv.4.1.1 package. No variation in or anomaly of the sella turcica was more frequent in CPO compared to non-cleft individuals. Patients with hypertrophic posterior clinoid process had higher interincisal and 1+:Nasion-A angles. Subjects with hypertrophic posterior clinoid process and double contour of the floor had higher Sella-Nasion-A, Sella-Nasion-B and Sella-Nasion-Pogonion and lower ANB. A pyramidal shape of the dorsum sellae was more prevalent in males, as was double contour of the floor in females. Subjects with an oblique anterior wall had lower SNB, GntgoAr and NLA. Subjects with a normal sella had higher SNPg, ML-NSL and 1+:NAmm. A normal sella was more prevalent in younger patients. CPO is not associated with sella turcica abnormalities compared to non-cleft orthodontic patients.

## 1. Introduction

In prenatal craniofacial development, the basal regions of the human skull undergo a series of complex and intricate processes. Initially, these parts of the skeletal system are established in a cartilaginous framework. Specifically, the hypophyseal cartilage, derived from both paraxial mesenchyme and neural crest cell populations, undergoes a process of endochondral ossification, leading to the formation of the postsphenoid part of the sphenoid bone. The paraxial mesenchyme contributes to the caudal part of the sella turcica, participating in the development of the rostral end of the notochord, whereas the neural crest cells contribute to the more rostral portion of the sella turcica and the prechordal skeleton. These two parts exhibit distinct molecular profiles and differentiation potentials, enabling them to contribute to specific regions. 

These developmental events are orchestrated through molecular and cellular interactions, involving the interplay of multiple signaling pathways and transcriptional regulatory factors [1]. 

The upper surface of the sphenoid bone is the sella turcica. This distinctive anatomical feature is divided into two parts. Its anterior slope bears a median tuberculum sellae, behind which is the concave hypophyseal fossa. The floor of the fossa is part of the roof of the sphenoidal sinuses, separated by a septum. Posterior to the fossa, the dorsum sellae projects upward and forward, contributing to the structure of the sella turcica. The sella turcica is completed and delimited laterally by two middle clinoid processes. The anterior border of the sella turcica is known as the tuberculum sellae. The superolateral angles of the dorsum sellae exhibit an expanded morphology, as the posterior clinoid processes, which serve as attachments for the tentorium cerebelli. On each side below the dorsum sellae, a small petrosal process articulates with the apex of the petrous part of the temporal bone. Posterior to the dorsum sellae, the sphenoid body slopes directly into the basioccipital bone, together forming the clivus [1,2]. 

The sella turcica serves as a crucial anatomical structure that hosts the pituitary gland, also known as the hypophysis. Any abnormality or pathology in the gland could manifest from an altered shape of the sella turcica. Disturbances in the regulation of glandular hormone secretion can result in a wide range of clinical manifestations and endocrine disorders, including the hypersecretion or hyposecretion of specific hormones, leading to hormonal imbalances with systemic effects on growth, metabolism, reproduction, and other physiological processes [2,3,4]. Moreover, it has been proven that abnormalities and pathologies in the pituitary gland may be associated with a low weight and a short stature, which are characteristic features within the phenotypic oculoauriculovertebral spectrum with radial defects [5,6].

Cephalometric radiographs of subjects affected by conditions caused by a disturbance in the regulation of glandular hormone secretion may, in some instances, reveal an abnormal sellar region, or vice versa; subjects with an abnormal sella turcica may in fact have an undetected underlying disease. A careful analysis of radiographic findings and correlation with clinical manifestations is crucial for identifying potential hormonal disorders and for ensuring appropriate diagnostic and management strategies [7,8,9]. Some researchers emphasize the important role of orthodontists in the initial diagnosis via imaging techniques. Surgical approaches continue to refine the knowledge and management strategies related to the pathologies of sella turcica, such as pituitary tumors and congenital malformations like clefts [1,2,9].

Abnormal neurocranial development has been observed in different craniofacial abnormalities. The most common craniofacial birth defects are orofacial clefts. Individuals affected by these conditions are characterized by impaired function of the masticatory system due to the disrupted growth of the facial skeleton. Among the most common challenges are difficulties with proper speech, swallowing and chewing. The sagittal facial disproportion in adolescents with a surgically operated cleft lip and palate is a well-known problem. Statistically significant differences have been found between the craniofacial morphology of bilateral cleft lip and palate (BCLP) and non-cleft patients, regardless of the number of surgical procedures and operators [10].

Few previous studies have been found to referr to the abnormalities of the sella turcica in patients with BCLP [11] and those with UCLP [11,12,13,14]. However, no studies have been found to specifically focus on sella turcica morphology in patients with cleft palate only (CPO).

The evaluation of sella turcica morphology and associated pituitary gland abnormalities plays a crucial role in the clinical assessment, diagnosis and management of endocrine disorders. Advanced and specialized imaging techniques, such as cephalometry or magnetic resonance imaging, enable the detailed analysis and diagnosis of sella turcica and hypophysis, aiding in the detection and characterization of various pathologies, such as pituitary adenomas, tumors, cysts and developmental anomalies. The early identification and appropriate treatment of sella-turcica-related abnormalities are essential in optimizing patient health, preventing diseases, as well as maintaining hormonal homeostasis [2,3,4]. A detailed understanding of the intricate anatomy and inter-relationships within this important region is of paramount importance.

The aim of the present study was to compare the prevalence of sella turcica abnormalities visible on cephalometric radiographs of patients with CPO versus a healthy, non-affected population. Moreover, the authors aimed to find out if there is any correlation between cephalometric craniofacial morphology or discrepancy and the type of sella turcica in patients with CPO. 

## 2. Materials and Methods

Eighty-nine cephalograms of patients with CPO were analyzed and compared to cephalograms of a matched control group of healthy orthodontic patients with no craniofacial deformities. The inclusion criteria applied for both groups comprised the following requirements: the good quality of the cephalograms, allowing the identification of the cephalomeric landmarks performed in patients aged between 4 and 40 years, and the patient being capable of undergoing radiological examination and skull X-ray imaging. Moreover, the inclusion criterion for the study group was a confirmed diagnosis of cleft palate based on clinical or imaging data. The exclusion criteria for both groups were as follows: significant developmental abnormalities other than orofacial clefts, diseases that might impact the craniofacial morphology and prior non-cleft surgical procedures on the skull. 

Sample size was verified using an online power and sample size calculator (surveysystem.com, accessed on 13 July 2023). At the level of clinical significance of 3 degrees for the angular cephalometric measurements and the confidence level of 95%, the sample size yielded 83. 

**Figure 1:**
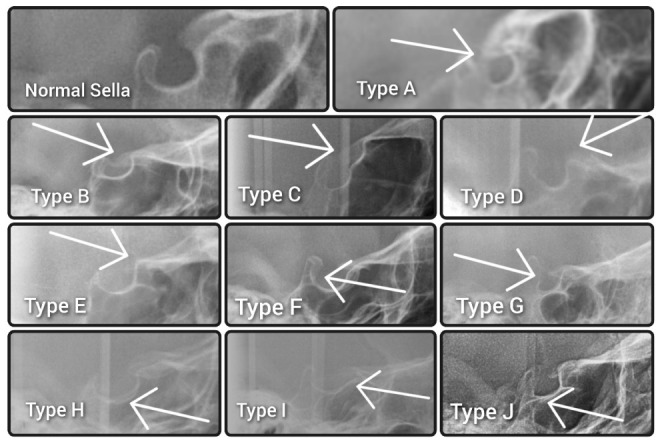
Morphology of normal sella turcica and types of abnormalities. Normal sella turcica. Type A—sella turcica bridge type A—ribbon-like fusion. Type B—sella turcica bridge type B—extension of the clinoid processes. Type C—incomplete bridge. Type D—hypertrophic posterior clinoid process. Type E—hypotrophic posterior clinoid process. Type F—irregularity (notching) in the posterior part of the sella turcica. Type G—pyramidal shape of the dorsum sellae. Type H—double contour of the floor. Type I—oblique anterior wall. Type J—oblique contour of the floor.

No cephalograms were made for the purpose of the study to avoid unnecessary excessive radiation. Ethical review and board approval have been waived for this study (decision reference No. KB-006/04/2022/Z). The morphologies of the sella turcicae were assessed according to the method described by Kucia et al. [15] and classified as either a normal sella turcica or ten variations, namely sella turcica bridge A—ribbon-like fusion; sella turcica bridge B—extension of the clinoid processes; C—incomplete bridge; D—hypertrophic posterior clinoid process; E—hypotrophic posterior clinoid process; F—irregularity (notching) in the posterior part of the sella turcica; G—pyramidal shape of the dorsum sellae; H—double contour of the floor; I—oblique anterior wall; or J—oblique contour of the floor, as presented in Figure 1. 

Each cephalogram was analyzed using the method described by Segner and Hasund [16] in specialized computer software (Ortodoncja 6.0, Orto-Bajt, Wroclaw, Poland). A detailed description of the measurements has been published in previous studies [15,16].

**Figure 2:**
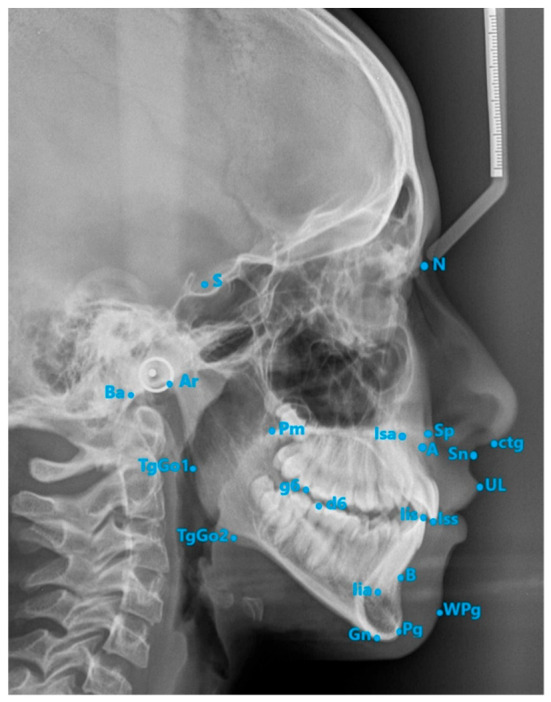
Cephalometric landmarks used. A—the deepest point on concave contour of anterior surface of the maxilla; Ar—construction point, where the lower border of cranial base crosses the posterior contour of mandibular ramus; B—the deepest point of anterior contour of the mandible; Ba—the most posterior and inferior point of the clivus; ctg—soft-tissue point on the curve, between the nasal base and prominence of the nasal tip; d6—distal cusp of lower first molar; g6—distal cusp of upper first molar; TgGo1—point of tangency of the line passing through Ar to the gonial region; TgGo2—point of tangency of the line passing through Gn to the gonial region; Iia—apex of the most protruded lower central incisor; Iis—incisal edge of the most protruded lower central incisor; Isa—apex of the most protruded upper central incisor; Iss—incisal edge of the most protruded upper central incisor; N—the most anterior point of frontonasal suture; Pg—the most prominent point of the chin; Pm—posterior nasal spine or the most inferior point of the mesial wall of pterygoid fossa; S—center of sella turcica; Sn—soft-tissue point between the nasal base and upper lip; Sp—anterior nasal spine; tgo—most distal point above the gonial angle; UL—most prominent point of the upper lip; WPg—most prominent point of the soft-tissue chin; Gn—gnation.

The cephalometric landmarks that were used are presented in Figure 2.

The initial analysis was performed by the primary and senior authors, and the consistency between examiners was confirmed using the intraclass correlation coefficient (ICC). The mean value of the two measurements was used for further comparisons and correlation analyses. Two weeks later, twenty-one cephalograms were randomly selected and reanalyzed by the same researchers to evaluate both inter- and intraexaminer reliability using ICC. The interpretation of the ICC values followed Cicchetti et al.’s (1994) guidelines [17]: ICC values above 0.75 indicated excellent reliability, ICC values between 0.6 and 0.75 indicated good reliability, ICC values between 0.4 and 0.6 indicated moderate reliability and ICC values below 0.4 indicated weak reliability between the measurements.

Statistical analysis was performed using the package R v.4.1.1 (IDE RStudio v. 1.4.1717). The level of statistical significance was set at α = 0.05. For variables on an interval scale, a description of the study set was made and the extraction of some basic conclusions and generalizations about the samples was carried out using grouped descriptive statistics. For this purpose, the describeBy() built-in method of the {psych} package was used. Variables on a nominal, ordinal scale were analyzed in pairs in the form of contingency tables with an indication of frequency.

The relationship of variables was examined using Pearson’s chi-square test or Fisher’s exact test. In addition, Cramer’s V measures of relationship strength were calculated (for this purpose, the tab_xtab() method of the {sjPlot} package was used).

In the presence of a significance test with the number of groups being more than two, the significance between pairs of groups was examined using a post hoc test (for this, the pairwiseNominalIndependence() method of the {rcompanion} package was used).

The hypotheses of Pearson’s chi-square test and Fisher’s exact test were as follows:

**H0:**  *The variables are independent; there is no relationship between the two nominal variables.*

**H1:**  *The variables are dependent; there is a relationship between the two nominal variables.*

To determine the correlation between a nominal (dichotomous) variable and a variable on an interval or quotient scale, a two-point correlation was calculated [18] via the cor.test() method of the {stats} package. If the correlation between two dichotomous variables was examined, a measure of the strength of the relationship phi was calculated using the phi() method of the {rcompanion} package.

## 3. Results

Eighty-nine cephalograms of CPO patients (including forty-seven females and fifty-two males) aged 4.55–37.61 years (mean age 12.65 years: nine patients younger than seven, sixty-nine patients between 7 and 18 and eleven patients over 18) were included in the study. The control group consisted of cephalograms of patients consecutively undergoing orthodontic treatment at the Department of Interdisciplinary Dentistry in Pomeranian Medical University in Szczecin aged 9.0–15.00 (mean age 12.15 years) (*n* = 89, including 48 females and 51 males). 

A high level of interexaminer reliability (between 0.813 and 0.979) was stated for all measurements according to the method used by Cicchetti et al. (1994) [19]. Moreover, when assessing intraexaminer reliability, the first author displayed excellent reliability in all measurements with a mean ICC of 0.954, while the other examiner demonstrated excellent reliability for most measurements according to the criteria used by Cicchetti et al. [19], with a mean ICC of 0.916. 

The distribution of sella turcica types in the study and control groups is presented in Table 1.

Fisher’s exact test did not reveal a statistically significant correlation between the type of sella turcica and CPO (*p* > 0.05).

**Figure 3:**
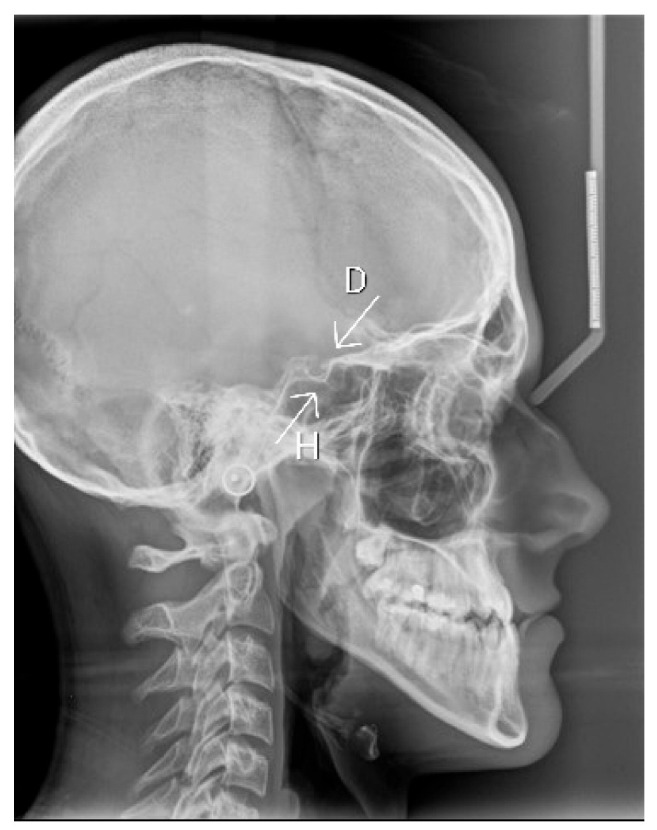
Cephalometric headfilm of a 20-year-old male patient from the study group with double contour of the floor of sella turcica (H) and hypertrophic posterior clinoid process (D).

It is interesting that in one patient, two anomalies of the sella turcica were found. The cephalometric headfilm of this subject is presented in Figure 3.

The distribution of cephalometric values in the study and control groups is presented in Table 2.

A significant positive correlation was found between hypertrophic posterior clinoid process and 1+:1 angle (*p* < 0.001). Moreover, a significant negative correlation was found with the 1+:NA angle (*p* = 0.013).

Hypertrophic posterior clinoid process with a double contour of the floor was associated with increased SNA (*p* = 0.036), SNB (*p* < 0.001) and SNPg (*p* < 0.001) angular values, as well as a reduced ANB angle (*p* = 0.013).

Statistically significant negative correlations were found between the oblique anterior wall and cephalometric values, including SNB (*p* = 0.034), GntgoAr (*p* < 0.001) and NLA (*p* = 0.008). 

Subjects with a normal sella turcica were characterized by higher values of SNPg (*p* = 0.046), ML-NSL (*p* = 0.041) angles and 1+:NAmm distance (*p* = 0.041).

Furthermore, the pyramidal shape of the dorsum sellae was found to be statistically significantly more often observed in males (*p* = 0.002), whereas the double contour of the floor was statistically significantly more prevalent in females (*p* = 0.005). The present study did not reveal any statistically significant sexual dimorphism in the prevalence of other sellar variations or anomalies.

What is more, a normal sella turcica was statistically significantly more prevalent in younger patients (*p* = 0.009).

## 4. Discussion

The formation processes of the sella turcica and the pituitary gland are inter-related. The anterior part of the sella forms from neural crest cells, whereas the posterior part is related to the notochord and develops from the para-axial mesoderm. These intricate developmental interactions contribute to the complex anatomical structure and functional relationship between the sella turcica and the pituitary gland [17,20].

A disturbance in this area (approximately at 7 weeks of gestation) may influence the morphology of the sella turcica. Any alteration or interference during this period can potentially impact the shape and structure of the sella turcica, leading to abnormalities or variations in its structure [21]. A relationship between cleft of the lip and palate and pituitary function is strongly suggested by numerous researchers [22,23,24,25]. Previous authors report a shorter stature in children with orofacial clefts, especially those involving the palate compared to their unaffected and healthy peers [26,27], suggesting pituitary insufficiency [22,23,24]. 

The sella turcica has been analyzed in malocclusions, as well. In the study by Alkofide [28], the sella turcica’s diameter was larger in Class III patients than in Class I subjects, while it was smaller in Class II subjects. Moreover, it has been found that abnormalities of the sella turcica are more prevalent in orthognathic patients compared to those treated by orthodontic means only [29].

In a study by Alkofide performed on 54 UCLP, 28 BCLP and 13 cleft-lip-only patients [30], the morphology of the sella turcica was altered in most subjects with clefts versus non-cleft individuals, especially in those with UCLP and BCLP. Moreover, the dimensions of the sella turcica were smaller in cleft subjects, especially with regard to the sellar depth in UCLP patients, but this increased with age, both in cleft patients and in non-cleft individuals. It can thus be noticed that sellar morphology and dimensions were more severely altered in patients with more severe forms of clefts.

The present paper is the first study referring to sella turcica abnormalities in CPO patients. Morphological abnormalities of sella turcica in cleft patients have been found by Alam and Alfawzam [11], who had stated a statistically significantly smaller distance between tuberculum sellae and posterior clinoid in UCLP, UCL and UCLA BCLP patients compared to non-cleft individuals. Moreover, the distance between the sella anterior and sella Posterior was smaller in patients with BCLP, UCLP and UCL. The distance between the tuberculum sellae and dorsum sellae was smaller in BCLP patients and lower in BCLP patients than in UCLP patients. In BCLP patients, it was smaller than in UCL patients. BCLP patients had a smaller tuberculum sellae–sella floor distance than unaffected controls, and BCLP patients had smaller distances than UCL patients. The distance between the posterior clinoid and sella floor was smaller in BCLP patients compared to unaffected controls and in BCLP vs. UCL patients. The sella median–sella floor distance was smaller in BCLP patients vs. controls and UCLP patients vs. controls. The sella area was smaller in BCLP and UCLP patients vs. controls. Thus, the most severe abnormalities were found in the most severe forms of orofacial clefts. This research sheds light on the distinctive sella turcica morphological variations observed in patients with cleft lip and palate. The findings suggest that these abnormalities are not only limited to the cleft itself but extend to the sella turcica region. The reduced distances and dimensions between specific sella turcica landmarks in cleft patients indicate altered growth and development patterns in this critical anatomical region [11]. 

These findings are consistent with correlations between sellar abnormalities and cephalometric values in the present study. What is more, these results emphasize the importance of further investigation into the underlying mechanisms that contribute to these sella turcica abnormalities in different forms of cleft palate and lip, in particular in cases of more severe cleft conditions. It seems that clinicians should consider abnormalities of the sella turcica when performing cephalometric analysis.

Referring to sexual dimorphism, the finding that the pyramidal shape of the dorsum sellae is found statistically significantly more often in males, whereas the double contour of the floor is more prevalent in females, is inconsistent with the study conducted by Kucia et al. [15], who did not find differences in sella turcica abnormalities between the sexes in children with malocclusion. Moreover, the present study revealed no statistically significant sexual dimorphism in the prevalence of sellar bridges, which is contrary to the studies by Axelsson et al. [31] as well as Kucia et al. [15]. Both studies reported sellar bridges to be more frequently observed in females. These conflicting results regarding sexual dimorphism in sella turcica morphology highlight the complexity of studying craniofacial variations. The differences observed between the studies suggest that additional factors beyond sex may contribute to the observed variations in sella turcica shape and structure. It is highly possible that the interplay of genetic, hormonal and environmental factors influences the development and presence of sella turcica abnormalities unequally in different populations.

The finding that a normal sella was significantly more prevalent in younger patients is inconsistent with the study by Jankowski et al. [32] on children (aged 6–15) with malocclusion. Contrary to the present study, papers including adults by Caderberg et al. [33], Arcos-Palomino and Ustrell-Torent [34] found that sellar bridges, which involve the fusion of the anterior and posterior clinoid processes, were age-dependent. This fact can be explained by the calcification of the sellar interclinoid and petroclinoid ligaments that occurs in adults [33]. As individuals age, these ligaments may undergo mineralization, leading to the formation of sellar bridges. The discrepancy in findings between the present study and previous research highlights the importance of considering age-related factors when studying sella turcica abnormalities. Future investigations should take into account the potential influence of age on the prevalence and development of sellar bridges to gain a comprehensive understanding of these anatomical variations in different age groups.

Moreover, it has to be noted that a bridge of the sella turcica, as observed on a 2D radiograph, can represent either a true union of the bones of the anterior and posterior process or may result from structures overlapping on a lateral cephalometric radiograph. Determining the exact nature of the observed bridge can be challenging due to the inherent limitations of two-dimensional imaging. The overlapping of anatomical structures and the projection angles can create the appearance of a bridge, making it difficult to accurately determine whether it signifies an actual fusion of bones or a mere radiographic overlap. Therefore, careful evaluation may be necessary.

Possible limitations of the present study refer to the two-dimensional cephalometric radiographs used. Since the ALARA rule was followed, cephalometric radiographs available in the patients’ records were used, and no radiographs were made for the purpose of the study in the patients included. Future studies referring to sella turcica abnormalities in patients with orofacial clefts could be based on CBCT, thus allowing a detailed analysis of the true sellar morphology, especially when referring to sellar bridges, and they could differentiate between a true union and the radiologic overlapping of bony structures. However, it must be kept in mind that recruiting a healthy control group could be extremely difficult, since CBCT is performed based on medical indications. It is not performed in healthy patients without any pathology within the facial bones. Whereas cephalometric radiographs are routinely taken in most orthodontic patients, the main purpose of referring an orthodontic patient for CBCT is to assess the 3D positions of impacted teeth. According to Jankowski et al. [32], abnormalities of the sella turcica are more frequent in patients with dental abnormalities than in unaffected orthodontic patients. Thus, patients with impacted teeth do not seem to constitute a proper control group when the analysis of sella turcica abnormalities is concerned.

## 5. Conclusions

1. Sellar abnormalities on cephalometric radiographs are not more prevalent in CPO patients than in non-cleft orthodontic patients.
2. Sella turcica abnormalities and craniofacial morphologies on cephalometric radiographs are interrelated, e.g., hypertrophic posterior clinoid process is associated with retruded incisors, whereas an oblique anterior wall is associated with a more retrusive mandible, a reduced gonial angle and a reduced nasolabial angle.

## Figures and Tables

**Table 1:** Distribution of the frequency of sella turcica abnormalities in the study (*n* = 89) and control groups (*n* = 89) and analysis of independence.

Sella Turcica Morphology	No in the Group			*df*	*χ* ^2^	*p*	*V*
	Study	Control	Total				
Sella turcica bridge type A—ribbon-like fusion	3	0	3	11	13.19	0.291	0.27
Sella turcica bridge type B—extension of the clinoid processes	2	1	3				
Incomplete bridge	9	6	15				
Hypertrophic posterior clinoid process (D)	8	5	13				
Incomplete bridge with pyramidal shape of the dorsum sellae (C)	1	0	1				
Hypotrophic posterior clinoid proces (E)	4	4	8				
Irregularity (notching) in the posterior part of the sella turcica (F)	8	9	17				
Pyramidal shape of the dorsum sellae (G)	2	5	7				
Double contour of the Floor (H)	5	4	9				
Oblique anterior wall (I)	4	0	4				
Oblique contour of the floor	2	1	3				
normal sella turcica (J)	41	54	95				

**Table 2:** Distribution of cephalometric values in the study and control groups.

Variable	Group	Mean	SD	Median	Min	Max	IQR	Skewness	Curtosis
Age	Study	12.65	5.56	11.61	4.55	37.61	6.16	1.70	4.46
	Control	12.15	1.71	12.0	9.0	15.0	2.00	0.18	−0.99
SNA	Study	78.22	4.87	78.50	65.10	88.80	6.20	−0.19	−0.08
	Control	80.41	3.58	80.30	71.40	90.50	4.20	−0.06	0.08
SNB	Study	76.50	5.08	76.60	65.40	94.10	6.70	0.44	0.49
	Control	77.20	3.57	77.50	68.00	85.20	4.30	−0.45	0.34
ANB	Study	1.71	3.97	1.70	−10.90	10.10	5.30	−0.32	0.09
	Control	3.22	3.19	3.50	−8.10	9.30	4.60	−0.63	0.55
SNPg	Study	77.34	5.23	77.50	64.40	95.60	7.70	0.33	0.73
	Control	78.02	3.57	78.40	68.40	85.50	4.30	−0.54	0.37
NSBa	Study	129.82	6.15	129.40	115.20	143.80	9.50	0.22	−0.59
	Control	130.24	4.41	130.00	120.40	138.70	6.30	−0.06	−0.75
GntgoAr	Study	131.95	8.53	132.20	104.00	150.80	10.90	−0.42	0.43
	Control	127.67	7.96	126.60	108.10	147.10	12.10	−0.11	−0.49
NL-NSL	Study	12.78	5.21	11.90	2.20	26.40	7.00	0.26	−0.46
	Control	8.44	3.63	8.20	1.10	21.40	4.50	0.78	1.31
ML-NSL	Study	37.96	8.07	37.00	23.20	63.70	10.90	0.59	0.04
	Control	34.31	6.06	34.40	19.10	48.80	7.30	0.13	−0.15
ML-NL	Study	25.18	7.00	24.60	11.20	47.30	7.90	0.60	0.32
	Control	25.88	6.33	26.50	7.70	45.20	7.30	0.14	0.57
H	Study	11.26	6.50	11.30	0.30	26.60	9.70	0.26	−0.77
	Control	13.35	5.79	13.40	0.00	28.90	6.60	−0.11	−0.03
1+:1- angle	Study	135.73	13.43	135.90	102.00	1.42	66.70	0.00	0.19
	Control	128.45	10.13	126.90	102.20	1.07	48.70	0.15	−0.19
1+:NA angle	Study	22.02	10.37	22.20	0.70	1.10	44.70	0.24	−0.25
	Control	23.23	6.89	23.50	9.00	0.73	39.00	0.58	1.18
1-:NB angle	Study	21.07	7.09	21.20	0.30	0.75	37.30	−0.20	0.03
	Control	25.09	6.83	25.20	8.20	0.72	37.30	0.26	0.50
NLA	Study	109.56	12.60	111.10	69.00	131.90	14.90	−0.77	0.73
	Control	115.28	9.75	116.20	80.00	135.30	9.40	−0.65	1.10
Pg:NB	Study	1.48	1.95	1.20	−3.30	7.90	2.50	0.70	1.07
	Control	6.71	7.00	4.90	−12.60	26.90	9.90	0.40	0.15
1+:NA	Study	2.47	3.52	2.40	−6.30	12.60	4.40	0.17	0.40
	Control	15.56	13.05	14.50	−19.70	48.90	15.50	0.30	−0.06
1-:NB	Study	2.67	2.65	2.20	−2.50	12.20	3.70	0.75	0.76
	Control	18.76	10.92	17.10	−3.50	50.20	12.70	0.41	0.22
Wits	Study	−2.22	3.95	−1.80	−14.90	5.60	5.90	−0.45	−0.02
	Control	−0.31	18.98	−1.60	−60.70	39.80	21.98	−0.59	0.90
Index	Study	79.22	7.66	79.90	58.10	96.00	9.30	−0.22	0.01
	Control	80.38	7.88	81.00	63.40	97.00	11.30	−0.07	−0.57

## Data Availability

All raw data are available upon request from corresponding author.
